# Editorial: Exploring the interaction between health-promoting and health risk behaviors in health, volume II

**DOI:** 10.3389/fpubh.2025.1668541

**Published:** 2025-08-01

**Authors:** Feng Jiang, Huixuan Zhou, Yi-lang Tang

**Affiliations:** ^1^School of International and Public Affairs, Shanghai Jiao Tong University, Shanghai, China; ^2^Institute of Healthy Yangtze River Delta, Shanghai Jiao Tong University, Shanghai, China; ^3^Institute of Health Policy, Shanghai Jiao Tong University, Shanghai, China; ^4^Department of Physical Fitness and Health, School of Sport Science, Beijing Sport University, Beijing, China; ^5^Key Laboratory of Exercise and Physical Fitness, Ministry of Education, Beijing Sport University, Beijing, China; ^6^Department of Psychiatry and Behavioral Sciences, Emory University School of Medicine, Atlanta, GA, United States; ^7^Mental Health Service Line, Atlanta VA Medical Center, Decatur, GA, United States

**Keywords:** physical activity, health promotion, health-risk behavior, lifestyle, interaction, health outcome

## 1 Introduction

The World Health Organization has highlighted that health-related behaviors can significantly influence wellbeing—either enhancing it or contributing to its decline (1). Based on this conceptual context, volume II of this Research Topic delves into the complex interactions between health-promoting and health-risk behaviors. This volume comprises 26 diverse studies, collectively addressing four interconnected themes: (1) managing health risks, (2) transforming health behaviors, (3) fostering supportive social and institutional networks for behavioral sustainability, and (4) coordinating health strategies across different life stages. Taken together, these studies signal a meaningful shift in perspective—from passively addressing symptoms to proactively managing health. This emerging approach emphasizes early intervention, empowering individuals with comprehensive capabilities, and aims to achieve better health outcomes through collective effort.

## 2 Managing health risks: from symptom control to systemic prevention

Several studies in this volume underscore a critical insight: health-risk and health-promoting behaviors often co-exist and exert independent effects on health outcomes. Studies of Li Z. et al., Bai et al., Zhang et al., Guo and Wang, Tian et al., challenged the long-held assumption that positive behaviors (such as physical activity) could offset the negative effects of a sedentary lifestyle (2). Instead, these findings reinforce the understanding that sedentary behavior and physical activity affect health through distinct mechanisms. On the other hand, a number of studies explored the concept of isochronous substitution–the idea that replacing one behavior with another can lead to different health outcomes (3). For example, Wang et al. identified non-linear interactions among behavioral clusters that contribute to obesity. Similarly, Liebig et al. showed that substituting screen time with outdoor or social activities could significantly improve health indicators among medical students. Collectively, these findings advocate for a paradigm shift in future health governance: from reactive, symptom-focused interventions to proactive, prevention-oriented strategies. This includes prioritizing behavioral design and prevention, addressing upstream determinants of health, embedding wellness into everyday environments, and fostering systems that support long-term behavioral change and sustained wellbeing.

## 3 Transformation of health behaviors: the cognitive–motivational chain

Behavioral change is not solely driven by knowledge and resources–it also hinges on an individual's intrinsic motivational state (4). A recurring theme in this volume is the significance of the cognitive-motivational chain in translating health intentions into sustained actions. Chen et al. utilized the COM-B (capability, opportunity, motivation, and behavior) model to highlight motivation as the key catalyst for behavioral change. Young-Silva et al. further demonstrated how self-efficacy and health literacy boost motivational engagement. Similarly, Liu et al. found that individuals who perceived themselves to be at higher health risks are more likely to adopt healthier diet and exercise habits.

Mental health also plays a pivotal role when it comes to behavioral transformation (5). Huangfu et al. discovered that adolescent body image anxiety is moderated by self-esteem and self-compassion, underscoring the importance of psychological resilience in maintaining behavioral goals. Kim and Shim emphasized the bi-directional relationship between mental health and perceived physical health, cautioning that neglecting emotional wellbeing may undermine the effectiveness of physical interventions.

Together, these studies suggest that individuals are not passive recipients of behavioral change. Their level of engagement depends on cognitive readiness (cognitive priming), meaningful motivation (motivation design), and emotional support (6).

## 4 Sustaining behavior through social and institutional scaffolding

For health-promoting behaviors to be sustained over time, they must be supported by a robust system of social and institutional scaffolding (7). Social scaffolding includes the influence of family, friends, or community networks, which may provide motivation, accountability, and emotional reinforcement. Institutional scaffolding, on the other hand, involves the role of schools, workplaces, healthcare systems, and governments, all of which may shape environments that enable and reinforce healthy behaviors. Together, these forms of social and institutional support create the conditions necessary for long-term behavioral sustainability.

Several studies in this volume emphasize how crucial social, cultural, and institutional networks are for maintaining health-promoting practices. Moise et al. highlighted how culturally embedded practices in the African-American community, such as herbal traditions, meditation, and religious rituals, serve as effective tools for improving sleep and managing chronic conditions. This underscores the essential role of cultural fitness in intervention design. Bamlaku Golla et al. showed that in rural Ethiopia, toilet use was more strongly influenced by social norms, economic conditions, and self-efficacy rather than health knowledge alone—revealing the limitations of didactic health education. The structural dimensions of behavior were further explored by Buková et al., who demonstrated how education and social structures shape health practices. Meanwhile, Li H. et al. showed through a randomized trial that medical institutions can act as platforms for digital empowerment and sustained engagement in health-promoting behaviors. Collectively, these research findings confirmed that sustainable health behaviors emerge not only from individual intention, but also from broader ecosystems—social, cultural, and institutional—that support and reinforce them.

## 5 Coordinated health governance across the life course

When viewed through a life course lens, the complexity of health behaviors becomes more apparent. Several studies in this volume advocate for an approach that considers behavioral patterns over time, rather than treating behaviors as isolated variables. For instance, Wang et al. found that combinations of behaviors, such as prolonged sedentary time, insufficient sleep, and exposure to second-hand smoke, significantly increased the risk of obesity compared to any single factor alone. This highlights the need for interventions that are designed to target specific behavioral clusters rather than individual behaviors in isolation. Buková et al. identified the transition from high school to college as a critical “behavioral cliff,” a period during which healthy habits often deteriorate. These results strengthen the importance of system-based interventions that respond to high-risk behavioral patterns, and are strategically timed to coincide with key turning points in life stages.

## 6 Conclusion: advancing toward a proactive health model

This volume brings together evidence from four key areas, offering more than a collection of isolated findings. Instead, the studies included in this volume reveal a set of converging insights that collectively signal a deeper theoretical evolution in understanding health behaviors. Central to this evolution is the growing recognition that health-related behaviors should not be treated as opposites or addressed in isolation. Rather, they interact dynamically across psychological, social, and institutional levels. This integrated perspective has given rise to a new paradigm—*proactive health*—in which the interplay between risky behaviors and health-promoting behaviors is not only acknowledged, but strategically leveraged to improve population outcomes (8, 9).

However, realizing this paradigm in practice requires more than conceptual clarity. Proactive health demands strategic design. If proactive health is the destination, we must chart the pathways to reach it. These pathways must address how behaviors interact at the micro-level (individual choices and motivations), how environments shape choices at the meso-level (community and institution settings), and how macro-level culture factors affect adoption and sustainability.

Three research directions merit particular attention. (1) Mechanism-oriented research: this approach uses causal models to show how behavioral clusters interact and reinforce one another, revealing the synergistic effects of combined behaviors. (2) Context-oriented strategies: these involve embedding behavioral change tools into real-world settings, such as schools, workplaces, and digital platforms, ensuring that interventions are practical and scalable. (3) Culturally oriented designs: tailoring interventions to align with local norms and values, and traditions enhances relevance and effectiveness, especially in diverse or underserved populations. Isolated actions or fragmented efforts will suffice. Proactive health requires a comprehensive framework that integrates risk reduction, behavioral support, and institutional empowerment. Only within this paradigm can health be transformed from a reactive, individual concern into a collaboratively cultivated public good.
